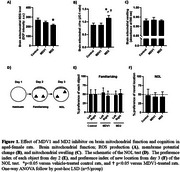# The Myeloid Differentiation Factor‐2 Inhibitor Alleviates Brain Mitochondrial Oxidative Stress Without Improving Cognitive Impairment in Aged‐Female Rats

**DOI:** 10.1002/alz70855_102430

**Published:** 2025-12-23

**Authors:** Hiranya Pintana, Patcharapong Pantiya, Chanisa Thonusin, Titikorn Chunchai, Suriphan Donchada, Busarin Arunsak, Guang Liang, Nipon Chattipakorn, Siriporn C Chattipakorn

**Affiliations:** ^1^ Neurophysiology Unit, Cardiac Electrophysiology Research and Training Center, Faculty of Medicine, Chiang Mai University, Chiang Mai, Thailand; ^2^ Center of Excellence in Cardiac Electrophysiology Research, Chiang Mai University, Chiang Mai, Thailand; ^3^ Office of Research Administration, Chiang Mai University, Chiang Mai, Thailand; ^4^ Department of Physiology, Faculty of Medicine, Chiang Mai University, Chiang Mai, Thailand; ^5^ Chemical Biology Research Center, School of Pharmaceutical Sciences, Wenzhou Medical University, Wenzhou, 325035, China, Zhejiang, China; ^6^ Department of Oral Biology and Diagnostic Sciences, Faculty of Dentistry, Chiang Mai University, Chiang Mai, Thailand

## Abstract

**Background:**

Therapeutic approaches that aimed to improve brain mitochondrial function, could potentially mitigate age‐related cognitive impairments. The myeloid differentiation factor‐2 (MD2) inhibitor, L6H2, has been shown to alleviate brain mitochondrial dysfunction and cognitive deficits in obese rats. Mitochondrial division inhibitor‐1 (MDV1) prevents excessive mitochondrial fission, promoting mitochondrial elongation, which has been reported to protect against neurotoxic damage. However, the effects of L6H2 and MDV1 on cognitive function and brain mitochondrial function in aged‐female rats remain unclear. Thus, this study aims to investigate whether MD2‐inhibitor (L6H2) or MDV1 attenuates cognitive impairment and brain mitochondrial dysfunction in aged‐female rats.

**Method:**

Female Wistar rats (*n* = 15, aged 24 months) were randomly divided into three groups: 1) vehicle‐treated as a control rats, 2) MD2 inhibitor‐treated rats, and 3) MDV1‐treated rats. Control rats received normal saline, MD2‐treated rats received L6H2 (40 mg/kg/day, p.o.), and MDV1‐treated rats were administered MDV1 (1.2 mg/kg/day, i.p.). After two‐weeks of daily treatment, cognitive function was assessed using the Novel Object Location (NOL) test. Brain mitochondrial function was measured after euthanasia.

**Result:**

Only MD2‐treated rats exhibited a significant reduction in brain mitochondrial reactive oxygen species (ROS) levels and an improvement in mitochondrial membrane potential, without affecting mitochondrial swelling, compared to the control group (Figure 1A‐C). There were no significant differences in preference index between the objects during the familiarization phase, indicating no object bias across groups (Figure 1E). In the NOL test, none of the groups exhibited a significant preference for the new location, as the preference index in all groups was not different from the fixed control value of 50%. This indicates cognitive impairment in the aged‐female rats, with neither treatment showing improvement. (Figure 1F).

**Conclusion:**

MD2‐inhibitor (L6H2) reduced brain mitochondrial ROS production and improved mitochondrial membrane potential change in aged‐female rats, but did not improve cognitive function or overall mitochondrial health. Additionally, MDV1 had no effect on mitochondrial function or cognitive outcomes. These results suggest that MD2‐inhibitor may play a role in reducing brain mitochondrial oxidative stress, the doses and treatment duration used in this study were insufficient to significantly improve cognitive function in aged‐female rats.